# Comparison of Cell Disruption Methods for Improving Lipid Extraction from Thraustochytrid Strains

**DOI:** 10.3390/md13085111

**Published:** 2015-08-11

**Authors:** Avinesh R. Byreddy, Adarsha Gupta, Colin J. Barrow, Munish Puri

**Affiliations:** Centre for Chemistry and Biotechnology, Geelong Technology Precinct, Deakin University, Waurn Ponds, Geelong 3217, Australia; E-Mails: arbyredd@deakin.edu.au (A.R.B.); adarsha.gupta@deakin.edu.au (A.G.); cbarrow@deakin.edu.au (C.J.B.)

**Keywords:** solvents, biodiesel, algae, DHA, marine, omega-3

## Abstract

Lipid extraction is an integral part of biodiesel production, as it facilitates the release of fatty acids from algal cells. To utilise thraustochytrids as a potential source for lipid production. We evaluated the extraction efficiency of various solvents and solvent combinations for lipid extraction from *Schizochytrium* sp. S31 and *Thraustochytrium* sp. AMCQS5-5. The maximum lipid extraction yield was 22% using a chloroform:methanol ratio of 2:1. We compared various cell disruption methods to improve lipid extraction yields, including grinding with liquid nitrogen, bead vortexing, osmotic shock, water bath, sonication and shake mill. The highest lipid extraction yields were obtained using osmotic shock and 48.7% from *Schizochytrium* sp. S31 and 29.1% from *Thraustochytrium* sp. AMCQS5-5. Saturated and monounsaturated fatty acid contents were more than 60% in *Schizochytrium* sp. S31 which suggests their suitability for biodiesel production.

## 1. Introduction

Biodiesel is emerging as a renewable and clean energy source to reduce carbon dioxide and greenhouse gas emissions. Biodiesel accounts for 10% of total biofuel production globally and its estimated production is about 6 billion L/year [1]. The present approach to biodiesel (defined as the monoalkyl esters of long-chain fatty acids) production involves transesterification of plant oils such as soybean oil, sunflower oil and rapeseed oil with methanol using alkali catalysts [2]. The use of plant oils for biodiesel production has been associated with some drawbacks such as high viscosity, low volatility and deposition in combustion chambers [3]. Also, the use of edible oils for biodiesel production has led to an increase in the price of oils, initiating a food versus fuel debate regarding biofuel sustainability. Feedstock useful for biofuel include soapstocks, acid oils, tallow oils, used cooking oils, various animal fats, non-edible plant oils and microbes including algae [4,5].

Microalgae are promising vehicles for the production of biodiesel and possess advantages such as higher growth rate and productivity, grow in various environments (fresh, brackish or salt water), do not compete for land, and have high oil productivity (20%–50% by dry weight basis) compared to conventional crops [6]. Selection of efficient microalgae species and suitable lipid extraction methods are important for commercial biodiesel production [7,8]. Biodiesel production from microalgae involves four major stages that are cultivation, cell harvest, lipid extraction and finally conversion of lipids into biodiesel [9]. Therefore, a suitable lipid extraction technique is a prerequisite for microalgal lipid extraction. Lipid extraction efficiency is dependent on the polarity of the solvent and combination of solvent mixture [9,10,11]. The combination of a polar and non-polar solvent mixture can in some cases extract more lipids from microalgae [12,13]. For example, the Bligh and Dyer method uses chloroform and methanol for lipid extraction from a range of biological samples [14]. The use of chloroform and ethanol in a 1:1 ratio provided maximum lipid extraction from *Chlorella* sp. [13], whereas, a combination of dichloromethane and ethanol increased lipid extraction efficiency by 25% in the same organism [15]. 

To get higher product recovery and quality lipids with lower operating costs from microbial cells, a suitable cell disruption method is required. Cell disruption enhances the release of intracellular lipids from microalgae by improving the access of the extracting solvent to fatty acids [16]. Cell disruption methods such as microwave, ultrasonication, bead mill, drying, and supercritical fluid extraction influence lipid extraction yields from a range of microalgae [7,9,17,18]. 

The aim of this study was to compare various organic solvents and cell disruption methods for effective lipid extraction from a new strain of thraustochytrids (a newly isolated strain from the Queenscliff region, Victoria, Australia). This strain was used as a representative microalgae in this work due to its ability to accumulate high levels of lipids [19]. In addition, microalgae biomass was used for different extraction methods that were successfully used for efficient algal lipid extraction in previous studies. Finally, the conversion of lipids to fatty acid methyl ester (FAME) was performed to determine yield and fatty acid distribution after extraction.

## 2. Results and Discussion

### 2.1. Biomass and Lipid Production

*Schizochytrium* sp. S31 showed highest biomass productivity at 0.81 g·L^−1^·day^−1^ and *Thraustochytrium* sp. AMCQS5-5 at 0.64 g·L^−1^·day^−1^ at the end of 5 days (Table 1). The lipid productivity of *Schizochytrium* sp. S31 and *Thraustochytrium* sp. AMCQS5-5 were 100.74 mg·L^−1^·day^−1^ and 64.2 mg·L^−1^·day^−1^, respectively. Selection of suitable microalgae strain with adequate biomass and oil productivity is important for cost effective biodiesel production. Our results on biomass and lipid productivity are in agreement with previous findings by Vello *et al.* (0.30 g·L^−1^·day^−1^ and 34.53 to 230.38 mg·L^−1^·day^−1^) that demonstrated the suitability of *Chlorella* strain as a promising candidate for biodiesel production [20]. 

**Table 1:** Biomass, lipid contents and productivity of thraustochytrids.

Properties	Thraustochytrid strains	
	*Schizochytrium* sp. S31	*Thraustochytrium* sp. AMCQS5-5
Dry weight (g·L^−1^)	4.06	3.23
Biomass productivity (g L^−1^·day^−1^)	0.88	0.64
Average lipid content (mg·L^−1^)	503.7	321.3
Lipid productivity (mg·L^−1^·day^−1^)	100.74	64.2

Lipid content and fatty acid profile of a microorganism is dependent on the growth conditions [21,22]. Medium composition influences the percentage of total lipid and type of fatty acids in the microbe. For example, the addition of Tween 80 in the production medium led to the accumulation of oleic acid in the thraustochytrids [23]. A recent study reported the effect of seasonal variation and nitrogen limitation in the total lipid production and fatty acid composition of *Nannochloropsis oculata*. They observed an increased accumulation (up to 90%) of saturated and mono-unsaturated fatty acids [24]. Since the aim of this work was to find a suitable disruption method for lipid extraction, higher biomass productivity was not pursued further. 

### 2.2. Lipid Extraction from Thraustochytrid by Organic Solvents

The lipid extraction by organic solvents was examined to confirm the lipid-extraction characteristics of thraustochytrids. Fatty acids present in the lipid govern the polarity, based on the principle “like dissolves like”, thus a suitable solvent should be identified for total lipid extraction, however a universal solvent cannot be applied to all microbes with different fatty acid composition. Total lipid extraction yields vary primarily with solvent polarity [25].

**Figure 1:**
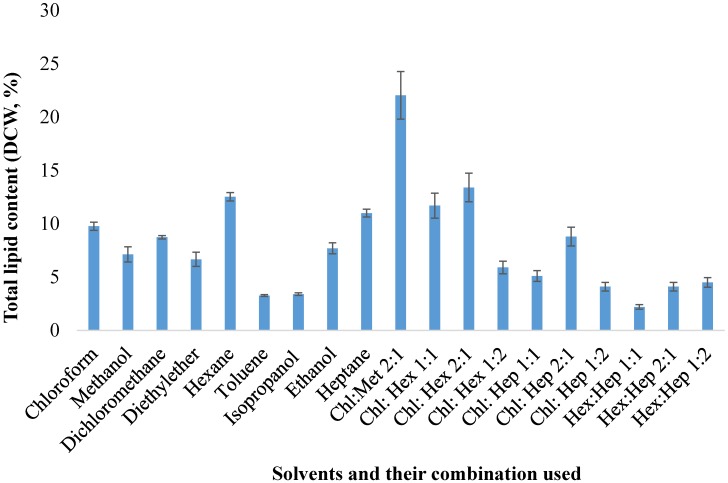
Lipid extraction percentage from dry biomass using various solvents from *Schizochytrium* sp. S31. The symbols represent Chl: choloroform, Hex: hexane, Hep: heptane, Met: methanol, DCW: dry cell weight.

To understand the efficacy of organic solvents in lipid extraction from *Schizochytrium* sp. S31, nine solvents and their combinations (based on high lipid yield) were selected, since the lipid extraction is highly dependent on the polarity of the solvents and their ratios [13]. The effect of single solvent as well as the combinations of the best 3 solvents with respect to the lipid extraction from *Schizochytrium* sp. S31 is demonstrated in Figure 1. Among the single organic solvents, maximum lipid (12.5%) was extracted with hexane, followed by 11% in heptane and 9.7% in chloroform. However, among the combination of solvents, the mixture of chloroform:methanol (2:1) showed maximum lipid extraction (22%), followed by chloroform and hexane (2:1) with 13.4% (Figure 1). Chloroform and hexane showed comparatively low efficacy in lipid extraction concluding that a range of polar to non-polar lipids were present in *Schizochytrium* sp. S31. A similar trend was observed during lipid extraction from *Chlorella* sp. [13]. A mixture of hexane:heptane (1:1) extracted the least amount of lipid (2.2%). Higher lipid yields with chloroform:methanol indicates the presence of more polar and neutral lipids in the algae. It was observed that the percentage of polar and neutral lipids were approximately 78% and non-polar lipids were 22% in some algaes [26]. Combination of polar and non-polar solvents could extract more lipids than individual solvents [10,12]. However, a study [27] contradicts this conclusion, depicting that combination of hexane and ethanol could not extract more lipid than hexane alone from *Scenedesmus dimorphus* and *Chlorella protothecoides*. This suggests the efficiency of lipid extraction depends on the algal species and their pre-existing lipid compositions. Also, results obtained from chloroform and methanol (2:1) specify the presence of more polar and neutral lipids in algae [13]. Mixtures of chloroform:methanol extract hydrocarbons, carotenoids, chlorophyll, sterols, triglycerides, fatty acids, phospholipids and glycolipids [28,29].

### 2.3. Comparison of Lipid Extraction Methods

**Figure 2:**
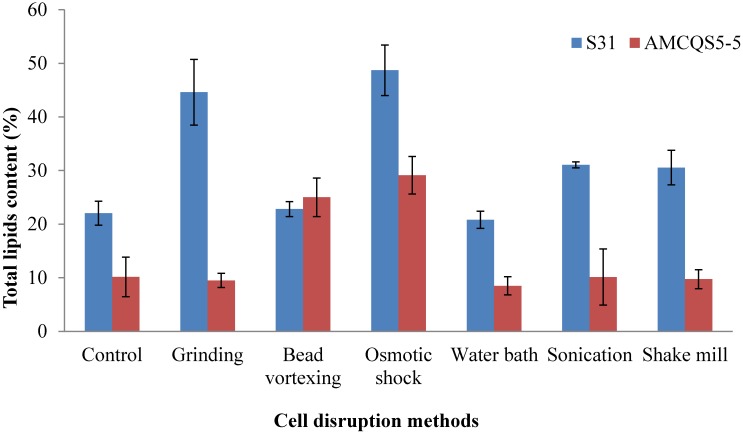
Effect of different cell disruption methods for lipid extraction from thraustochytrids. Average lipid extraction of each method was reported as % of total lipids extracted.

Six methods were evaluated in order to understand the efficiency of cell disruption methods for total lipid extraction from thraustochytrids. The effectiveness of the cell disruption methods were determined using lipid yield percentages. Cell disruption breaks the cells and improves the accessibility to the intracellular components for extraction [30]. Figure 2 shows the percentage of total lipids extracted as a function of cell disruption methods. All the cell disruption methods used in this study were able to disrupt thraustochytrid cells, although lipid yield varied. 

**Figure 3:**
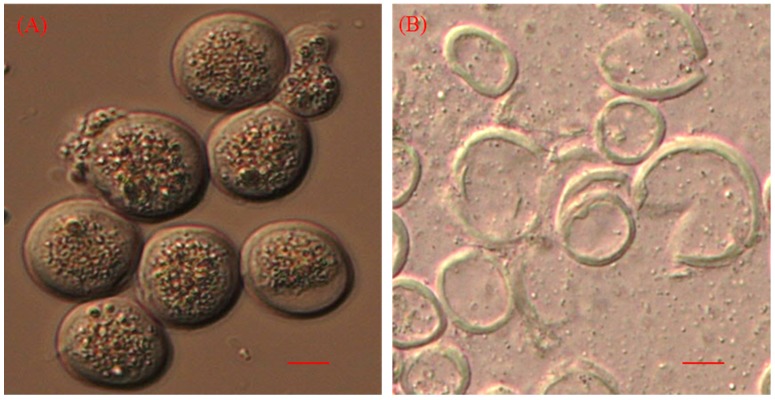
Effect of cell disruption on thraustochytrids cells. Thraustochytrid cells before (**A**), and after (**B**) cell disruption (scale bar 20 µm).

Thraustochytrid cells subjected to disruption resulted in rupture of the cell walls and release of intracellular components (Figure 3A,B). It was observed that lipid content of the *Schizochytrium* sp. S31 was higher than *Thraustochytrium* sp. AMCQS5-5. Maximum lipid was extracted from both *Schizochytrium* sp. S31 (48.7%) and *Thraustochytrium* sp. AMCQS5-5 (29.1%) cells using osmotic shock (Figure 2). Grinding, sonication, shake mill and water bath treatments extracted 44.6%, 31%, 30.5% and 20.8% of lipids, respectively. Bead vortexing resulted in 25% lipid extraction from *Thraustochytrium* sp. AMCQS5-5 cells, whereas other methods (water bath, grinding, shake mill and sonication) resulted in lower yields. Osmotic shock resulted in a 2.2-fold increment in lipid extraction from *Schizochytrium* sp. S31 and a 2.8-fold increase from *Thraustochytrium* sp. AMCQS5-5, compared to control. A similar study carried on *Chlorella* sp. indicated that osmotic shock an effective method for extracting lipids [31]. This method also consumes less energy than traditional methods. In a recent study, when *Chlamydomonas reinhardtii* cells were incubated in a high osmotic environment, it led to a 2-fold improvement in lipid extraction [32]. Table 2 summarises some cell disruption methods reported for lipid extraction from microalgae. Due to differences in cell wall structure, not all microalgae respond the same to pretreatment. For example, Lee *et al.* observed that microwave treatment was optimum for the disruption of *Botryococcus* sp., *Chlorella vulgaris* and *Scenedesmus* sp. cells [9]. Another study showed that grinding with liquid nitrogen facilitated higher levels of lipid extraction from *Chlorella vulgaris* [17]. Available literature suggests that cell disruption methods improve lipid extraction from microalgae, it depends on microalgae species, age of the culture and composition of cell wall. Therefore, results obtained from one species cannot be generalised to all other species [16]. 

**Table 2:** Comparison of cell disruption methods employed to extract total lipids from different microalgae.

No.	Cell disruption methods used	Efficient method	Organisms used	Lipid content (%)	Reference
1	Autoclaving	Microwaves			[9]
	Bead beating		*Botryococcus* sp.	28.6	
	Microwaves		*Chlorella vulgaris*	11	
	Sonication		*Scenedesmus* sp.	11.5	
	Osmotic shock				
2	Sonication	Sonication			[31]
	Osmotic shock		*Chlorella* sp.	20.1	
	Microwave		*Nostoc* sp.	18.2	
	Autoclave		*Tolypothrix* sp.	14	
	Bead beating				
3	Grinding	Grinding	*Chlorella vulgaris*	29	[17]
	Sonication				
	Bead milling				
	Enzymatic lysis				
	Microwaves				
4	Grinding	Osmotic shock			This study
	Bead vortexing		*Schizochytrium* sp. S31	48.7	
	Osmotic shock				
	Water bath		*Thraustochytrium* sp. AMCQS5-5	29.1	
	Sonication				
	Shake mill				

There are many lab scale cell disruption methods as discussed in the literature, however, only few mechanical methods either alone or with the intervention of enzymes/chemicals can be scaled up for industrial applications. For instance, bead mill, high pressure homogenizer and Hughes press are used extensively at large scale, which reduce unit operation steps compared to chemical and enzymatic methods [33,34]. Osmotic shock method was implemented in thraustochytrid cell disruption and lipid extraction, to reduce the energy consumption and production cost. During the osmotic shock treatment, the resulting wastewater can be recycled through reverse osmosis technology [35]. Same method has been applied at pilot-scale for enhancing the release of ectoine [36]. A recent study by Jayaranja and Rekha presented that the osmotic shock was most suitable method in extracting intracellular products, which can also be industrially scaled up [37]. The advantages and disadvantages of selected investigated methods are summarised in Table 3.

**Table 3:** Advantages and disadvantages of the investigated cell disruption methods.

Cell disruption methods	Advantages	Disadvantages
Manual grinding	- Quickest and efficient - 2 min process	- Localised heating caused denaturation of molecules
Bead vortexing	- Can be established easily and relatively effective	- High heat generation, - Incomplete cell lysis
Osmotic shock	- Lower energy consumption - Easier scale-up	- Generation of waste salt water - Time consuming
Water bath	- Maximum disruption - Easy in handling at lab scale	- Increases the viscosity - Energy intensive
Sonication	- Faster extraction - Suitable for all cell type	- Damage chemical structure of molecules
Shake mill	- Rapid method	- High energy intensive - High heat generation

### 2.4. Fatty Acid Composition of Extracted Lipid

**Figure 4:**
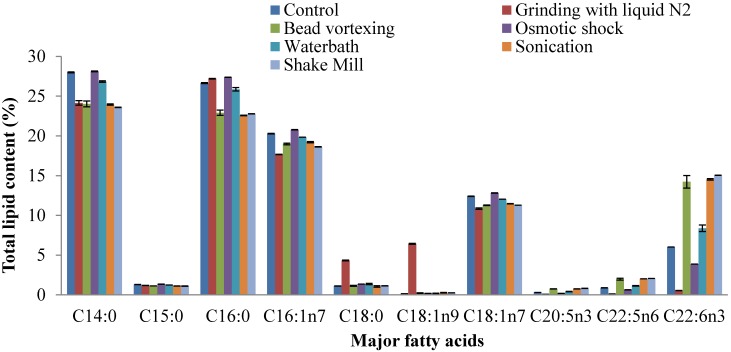
Effects of different cell disruption methods on fatty acid profiles of *Schizochytrium* sp. S31.

**Figure 5:**
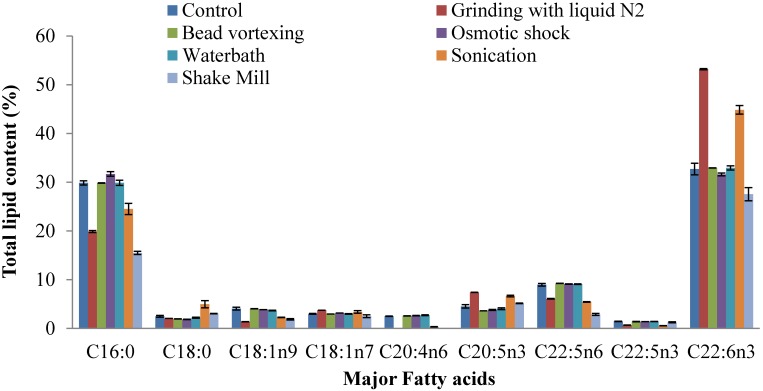
Effects of different cell disruption methods on fatty acid profiles of *Thraustochytrium* sp. AMCQS5-5.

**Figure 6:**
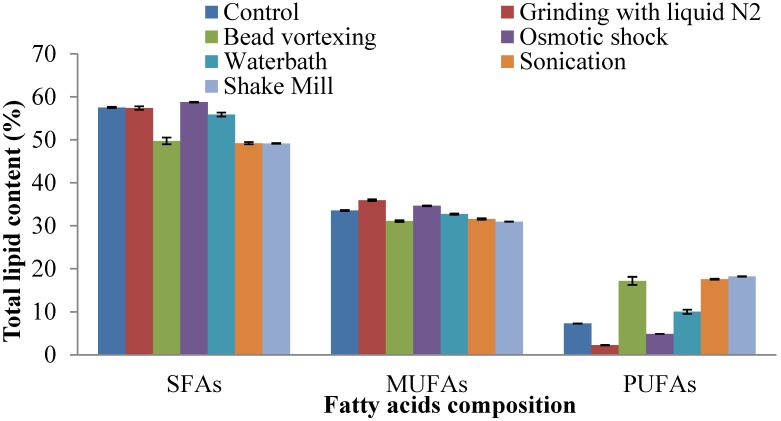
Effects of different cell disruption methods on saturated fatty acids (SFAs), monounsaturated fatty acids (MUFAs), and polyunsaturated fatty acids (PUFAs) of *Schizochytrium* sp. S31.

The fatty acid profiles of the lipids extracted following different cell disruption methods from thraustochytrids are presented in Figure 4 and Figure 5. Major fatty acids such as myristic acid (28.1%), palmitic acid (27.3%), palmitoleic acid (20.7%) and oleic acid (12.8%) were detected in *Schizochytrium* sp. S31 (Figure 4) based on osmotic shock cell disruption. Total saturated, monounsaturated and polyunsaturated fatty acid contents of osmotic shock method were 58.7%, 34.6% and 4.8%, respectively, making it a potential feedstock for biodiesel production (Figure 6). The other prominent fatty acids based on grinding, sonication and shake mill identified in the lipid extracts were saturated (49%–57%), mono-unsaturated (31%–35%) and polyunsaturated fatty acids (2%–18%). Saturated and monounsaturated fatty acids are useful major components for microalgal biodiesel production because of their relatively high oxidative stability [38]. The general properties of biodiesel such as viscosity, specific gravity, cetane number, iodine value, and low temperature performance metrics are determined by the structure (length and unsaturation) of fatty acid esters [39].

**Figure 7:**
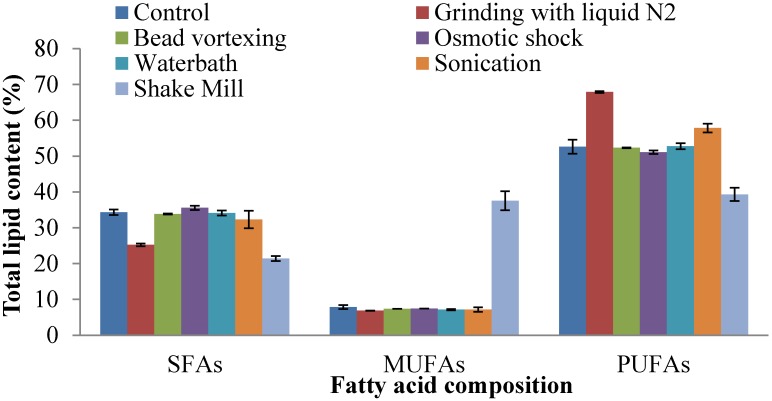
Effects of different cell disruption methods on saturated fatty acids (SFAs), monounsaturated fatty acids (MUFAs), and polyunsaturated fatty acids (PUFAs) of *Thraustochytrium* sp. AMCQS5-5.

In *Thraustochytrium* sp. AMCQS5-5, palmitic acid (31.6%) and docosahexaenoic acid (31.5%) were the major fatty acids (Figure 5), with other polyunsaturated fatty acids (C20:4*n*6, C20:5*n*3, C22:5*n*6 and C22:5*n*3) ranging from 2% to 9%, when cells were disrupted using osmotic shock. Saturated, monounsaturated and polyunsaturated fatty acid contents were 35.5%, 7.4% and 51%, respectively (Figure 7). The highest polyunsaturated fatty acid percentages were extracted by grinding and sonication methods, 67% and 57%, respectively. Only shake mill resulted in a significant percentage of monounsaturated fatty acids extraction (37.5%). Most methods resulted in different lipid yields, but no difference in the fatty acid profiles for extraction from *Thraustochytrium* sp. AMCQS5-5 (Figure 7). If the omega-3 fatty acids could be separated from other fatty acids in the *Thraustochytrium* sp. AMCQS5-5 extract, then these fatty acids, particularly DHA (docosahexaenoic acid), could be used as nutritional products and offset the cost of biofuel production in this strain [40,41].

### 2.5. Prediction of Biodiesel Properties

We analysed fatty acid profiles of two different thraustochytrid strains to understand the suitability of microalgae for biodiesel production. The fatty acid values were taken as an input in predicting the biodiesel properties by using an open access software Biodiesel Analyzer^©^ Ver. 1.1 [42]. Some of the important parameters of biodiesel are cetane number (CN), Iodine value (IV) and oxidative stability (OS), which determine the combustion behaviour, quality of biodiesel, stability and performance, respectively [43]. The CN and IV values in *Schizochytrium* sp. S31 (47.4 and 98.61) when compared to *Thraustochytrium* sp. AMCQS5-5 (44.01 and 157.44) were observed. According to ASTM D6751-12 (American Society for Testing and Materials, for standards and specifications for biodiesels) , the standard values were 47–51 (CN) and 120 g I_2_/100 g maximum, indicating suitability of selected strain S31 for biodiesel; however, its further characterization will be a follow up study. The OS value of 6.5 for *Schizochytrium* sp. S31 was higher than that of 1.65 for *Thraustochytrium* sp. AMCQS5-5, suggesting oxidation stability decreased with the increase of polyunsaturated fatty acid content. Other properties such as saturated fatty acids (SFA), monounsaturated fatty acids (MUFA), polyunsaturated fatty acids (PUFA), degree of unsaturation (DU), saponification value (SV), long chain saturated fatty (LCDF), cold filter plugging point (CFPP), cloud point (CP), allylic position equivalent (APE), bis-allylic position equivalent (BAPE), oxidation stability (OS), higher heating value (HHV), kinematic viscosity (μ), and density (ρ) were analysed as summarised in Table 4. 

**Table 4:** Biodiesel properties of the given thraustochytrids strains.

Properties	Units	Strain S31	Strain AMCQS5-5
Saturated fatty acid	% (m/m)	59	44
Monounsaturated fatty acids	% (m/m)	35	4.6
Polyunsaturated fatty acids	% (m/m)	14	33
Degree of unsaturation		63	70
Saponification value	mg KOH/g oil	233.98	164.73
Iodine value	g I_2_/100 g	98.61	157.44
Cetane number	min	47.44	44.01
Long chain saturated factor	% (m/m)	3.8	5.85
Cold filter plugging point	^o^C	4.54	1.90
Cloud point	^o^C	9.74	10.79
Allylic position equivalents		83	167.60
Bis-allylic position equivalents		70	165.20
Oxidation stability	h	6.5	1.65
Higher heating value	^o^C	42.14	32.33
Kinematic viscosity	mm^2^/s	1.28	0.94
Density	kg/m^3^	0.94	0.73

### 2.6. Energy Analysis

Energy consumption of the investigated cell disruption methods was attempted to understand their potentialities in large scale process. The comparative estimated energy consumptions and processing times of the investigated cell disruption methods are presented in Table 5. In comparison, water bath and sonication methods resulted in highest energy consumption, 2400 MJ·kg^−1^ dry mass and 1200 MJ·kg^−1^ dry mass, respectively. Shake mill energy consumption was estimated to be 690 MJ kg^−1^ dry mass. Osmotic shock method consumed modest energy (4.8 MJ·kg^−1^) followed by highest lipid recovery, thus preferred as choicest method. Whereas, the NaCl present in the lysate solution exerted osmotic pressure which was estimated to be 4.21 kPa using online osmotic pressure calculator. A recent work showed that the osmotic pressure 1.9 kPa was enough to break the microbial cells [37]. It has been demonstrated that energy consumption for the microwave (28 MJ·Kg^−1^ ) and ultrasound (44 MJ·kg^−1^) method enhanced lipid extraction from *Chlorella* sp. [44], which indicated that one of the investigated methods osmotic shock consumed less energy.

**Table 5:** Energy consumption comparison.

Cell Disruption Methods	Lipid Yield (%)	Energy Consumption (MJ·kg^−1^ Dry Mass)	Processing Time (min)
Control	22.04	Nil ^a^	0
Manual grinding	44.6	ND ^b^	2
Bead vortexing	22.8	48	20
Osmotic shock	48.7	4.8 ^c^	2
Water bath	20.8	2400	20
Sonication	31.05	1200	20
Shake mill	30.5	690	5

Thraustochytrids mass concentration of 16.6 kg/m^−3^ was used for energy analysis. Nil ^a^ represents no energy was consumed; ND ^b^ represents physical effort cannot be quantified; ^c^ Osmotic pressure by virtue of salt addition.

In this study, we have shown that some cell disruption methods, particularly osmotic shock, result in both different oil yields and variation in the percentage of saturated fatty acids, monounsaturated fatty acids and polyunsaturated fatty acids in the extracted oil. 

## 3. Experimental Section 

### 3.1. Chemicals

All the chemicals used in this study were of analytical grade. Medium components such as glucose, yeast extract and mycological peptone (Sigma-Aldrich, St. Louis, MO, USA) and sea salt (Instant Ocean, Blacksburg, VA, USA) were used for biomass production, while solvents such as acetone, ether, hexane (Merck, Sydney, NSW, Australia), methanol and ethyl acetate (Fischer and Honeywell, Melbourne, VIC, Australia) were used for lipid extraction. 

### 3.2. Strain Selection and Biomass Production 

*Schizochytrium* sp. S31 (ATCC 20888) was procured from American Type Culture Collection (ATCC) and used as standard culture. Thraustochytrids used in this study were maintained on GYP (Glucose, yeast extract and peptone) medium consisting of (g·L^−1^): glucose 5, yeast extract 2, mycological peptone 2, agar 10 and artificial seawater 50% at 25 °C and sub-cultured for 15 days.

*Thraustochytrium* sp. AMCQS5-5 (an in house isolate; GenBank accession number JX993841), was grown in a medium containing (g·L^−1^): glucose 5, peptone 2, yeast extract 2 and artificial sea water 50% for inoculum preparation with shaking at 150 rpm for 2 days at 25 °C. The medium was autoclaved at 121 °C for 20 min. Inoculum (5% *v*/*v*) was used to inoculate production medium (100 mL contained in 500 mL flask) and incubated for 5 days in a shake flask at 25 °C and 150 rpm. The resultant biomass was harvested by centrifugation (4000 rpm for 15 min) and was freeze-dried and kept at −20 °C until further use. 

The thraustochytrids grown in culture medium were harvested at the interval of 24 h up to 120 h. Optical density at 600 nm and dry cell weight (DCW) was measured at 24 h intervals. A calibration curve was plotted between OD and dry cell weight. Results are presented as mean ± standard deviation (SD) of duplicates repeated twice. The biomass and lipid productivity was calculated from the formula mentioned below:

Productivity = Biomass or Lipid content/(T_1_ − T_0_)

where, T_1_ = Final day of biomass harvesting and T_0_ = Initial day of incubation.

### 3.3. Lipid Extraction from Thraustochytrids by Organic Solvents

The nine solvents chloroform, dicholoromethane, diethylether, ethanol, heptane, hexane, isopropanol, methanol, toluene at ratios of 1:1, 1:2 and 2:1 were tested for maximising extraction of total lipids. For solvent extraction, 50 mg of freeze dried biomass of thraustochytrid was blended with 3 mL of various solvents. The mixture was vortexed for 2 min and the sample was then centrifuged at 4000 rpm for 15 min. Supernatant (organic phase) was carefully collected in the pre-weighed glass vials and the solvent was evaporated under nitrogen gas at room temperature. Lipid content (% dry weight basis) was determined gravimetrically. To determine the optimal organic solvent mixture for lipid extraction from thraustochytrids, different ratios of the best three single solvents were investigated. For chloroform-methanol, the Bligh and Dyer method was followed [45].

### 3.4. Cell Disruption for Lipid Extraction

Freeze-dried biomass was blended with 3 mL of chloroform and methanol (2:1) and disrupted by means of different cell disruption methods as detailed below. After each treatment, lipid extraction from thraustochytrids was done according to Gupta and co-workers [46]. After centrifugation (4000 rpm for 15 min), the upper layer was collected and dried under nitrogen gas. Lipid content (% dry weight basis) was determined gravimetrically.

#### 3.4.1. Grinding with Liquid Nitrogen

A sample of freeze-dried thraustochytrid biomass (50 mg) was taken in the ceramic mortar. About 10–15 mL liquid nitrogen was added and the sample was allowed to thaw and grinded with pestle for 2 min. After grinding the lipid was extracted using organic solvents.

#### 3.4.2. Bead Vortexing

Thraustochytrid cell suspension (50 mg) was taken in glass tube (35 mL) and 3 mL of solvent and 1 mL of beads (zirconia beads, size 0.4–0.6 mm, Klausen Pty Ltd., Blaxland, NSW, Australia) were added and contents were vortexed for 20 min, using a vortex in 30-second bursts. Samples were kept on ice in between the bursts and lipid was extracted using organic solvents.

#### 3.4.3. Sonication

Thraustochytrid cell biomass (50 mg) was suspended in 3 mL of solvent in a 15-mL centrifuge tube. Sample was sonicated at 20 kHz, 40% amplitude and the pulse was 40 s on and 20 s off with total working time of 20 min (Sonics, Newtown, CT, USA). Sample tubes were kept on ice during the sonication process to prevent overheating. 

#### 3.4.4. Osmotic Shock

Thraustochytrid suspension was disrupted using osmotic shock method. 50 mg of thraustochytrid biomass was suspended in 3 mL of 10% NaCl solution (mass concentration 16.6 kg/m^−3^, 0.6 kg of NaCl) and vortexed for 2 min and incubated for 48 h at room temperature, followed by solvent extraction. 

Cell disrupting pressure was calculated based on Morse equation:

π = *i*MRT

where π is cell disrupting pressure generated due to osmotic shock (units in atm or kPa), *i* is dimensionless van’t Hoff factor, *M* is molar concentration of NaCl, *R* is 0.0821 L·atm·K^−1^·mol^−1^, and *T* is absolute temperature in K. Osmotic shock was calculated using an online osmotic pressure calculation hosted by Georgia State University [47].

#### 3.4.5. Water Bath

3 mL sample containing 50 mg of biomass taken in a 15-mL centrifuge tube was placed in preheated water bath (Ratek Instruments Pty Ltd., Boronia, VIC, Australia) to induce the thermolysis. Samples were kept in water bath for 20 min at 90 °C. Three tubes were treated simultaneously as replicates without shaking.

#### 3.4.6. Shake Mill

Thraustochytrid cell suspension was disrupted in plastic sample bottle using shake mill (SPEX Mill 8000M, Metuchen, NJ, USA). The biomass and bead ratio was 3:1 ratio (Zirconia beads 0.4–0.6 mm) at maximum speed (1060 cycle/min) and exposed for 5 min.

All the cell slurries were observed under the microscope using differential interference contrast (Axio-imager, Zeiss, Oberkochen, Germany) to check the disruption of thraustochytrid cells. Microbial smear was prepared on the glass slide, air dried and observed under a microscope. All of the experiments were performed three times.

### 3.5. Fatty Acids Methyl Esters (FAMEs) Analysis

Fatty acids were converted to methyl esters by acid-catalysed trans-esterification according to the method [48]. 1 mL toluene was added to the glass tubes followed by the addition of 200 μL of internal standard, methyl nonadecanoate (C19:0) and 200 μL of butylated hydroxytoluene (BHT). Acidic methanol (2 mL) was also added to the tube and kept for overnight incubation at 50 °C. Fatty acid methyl esters (FAMEs) were extracted into hexane. The hexane layer was removed and dried over sodium sulphate. FAMEs were concentrated using nitrogen gas. The samples were analysed by a gas chromatography-flame ionization detector (GC-FID) system (Agilent Technologies, 6890N, Santa Clara, CA, USA). The GC was equipped with a capillary column (SGE, BPX70, 30 m × 0.25 mm, 0.25 μm thickness). Helium was used as the carrier gas at a flow rate of 1.5 mL·min^−1^. The injector was maintained at 250 °C and a sample volume of 1 μL was injected. Fatty acid peaks were identified on comparison of retention time data with external standards (Sigma-Aldrich) and corrected using theoretical relative FID response factors [49]. Peaks were quantified with Chemstation chromatography software (Agilent Technologies). Results are presented as mean ± SD of triplicates.

## 4. Conclusions 

This study has investigated the efficacy of various solvents on lipid extraction from *Schizochytrium* sp. S31. Chloroform, hexane, and heptane resulted in the highest lipid yields from the individual solvents tested. However, solvent combinations gave higher yields, with chloroform-methanol giving the highest lipid yield of 22%. Cell disruption methods further increased lipid recovery from this strain, using the optimised chloroform and methanol solvent combination. Osmotic shock resulted in a 2-fold increase in lipid yield when compared with solvent alone. Fatty acid analysis of *Schizochytrium* sp. S31 oil showed high levels of saturated and monounsaturated fatty acids, indicating a composition potentially useful for biofuel production. Predicted biodiesel properties also confirms suitability of *Schizochytrium* sp. S31 for biodiesel production. *Thraustochytrium* sp. AMCQS5-5 produced relatively high levels of PUFAs and so may have better utility in nutritional applications rather than as a biofuel producing strain. 
